# Klippel-Trenaunay Syndrome: To Be or Not to Be Afraid

**DOI:** 10.7759/cureus.52361

**Published:** 2024-01-16

**Authors:** Gautam N Vekariya, Sudhir Singh, Sabha Neazee, Sugat Jawade, Amey R Gujrathi

**Affiliations:** 1 Dermatology, Venereology, and Leprosy, Datta Meghe Medical College, Datta Meghe Institute of Higher Education and Research, Nagpur, IND; 2 Dermatology, Venereology, and Leprosy, Jawaharlal Nehru Medical College, Datta Meghe Institute of Higher Education and Research, Wardha, IND

**Keywords:** sclerotherapy, bone and soft tissue hypertrophy, macrodactyly, port wine stain, capillary-lymphatic-venous malformation, klippel-trenaunay syndrome

## Abstract

Klippel-Trenaunay syndrome (KTS) is a rare genetic syndrome comprising an abnormal development of soft tissues and the lymphovascular system with bony overgrowth, venous malformation, and port wine stains. We present an interesting case of a three-year-old child brought to our hospital with a swollen limb and raised skin lesions associated with bleeding from minor trauma. Most of the clinical characteristics of KTS were seen in our patient, including arteriovenous, soft tissue, capillary, and lymphatic abnormalities. The diagnosis of KTS is based on clinical examinations and imaging investigations. He had gross hypertrophy of the left lower limb with measurable lengthening compared to the opposite limb. Ultrasonography of the left limb revealed soft tissue hypertrophy with abnormal venous communication. The management of KTS is mainly symptomatic and should be approached conservatively if the patient has functional limbs without edema, bleeding, ulceration, or pain.

## Introduction

Klippel-Trenaunay syndrome (KTS), also known as capillary lymphatic-venous malformation (CLVM), is a rare congenital disorder, and the incidence is approximately 1:100,000 births [1]. KTS is a complicated vascular anomaly characterized by bone and soft tissue overgrowth, capillary malformations, and venous and lymphatic abnormalities [2]. A nevus flammeus, also known as a port wine stain, is a type of cutaneous capillary defect that appears in the extremities at birth [3]. Some patients may develop hemorrhagic papules or plaques along with varicose veins [4]. Vascular abnormalities develop mainly in the lower extremities [5]. KTS can be easily diagnosed at birth by its classical clinical presentation, but older age groups can have KTS if it is not detected at birth. KTS is a low-flow type of vascular malformation and is different from Klippel-Trénaunay-Weber syndrome, which is a high-flow defect that occurs due to arteriovenous malformation [6], consisting of the characteristics of KTS with arteriovenous fistula. We present the case of a male child who was brought to the hospital due to bleeding from leg lesions later diagnosed as Klippel-Trénaunay syndrome.

## Case presentation

A three-year-old boy from a village was taken by his parents to a tertiary care hospital in central India with complaints of raised skin lesions on the left limb and a discrepancy in the size difference between the two lower limbs. The lesions were present since birth and were associated with occasional bleeding on minor trauma. On a detailed physical examination, his pulse was 88 beats per minute. He was normothermic. He had pallor without visible jaundice, cyanosis, clubbing, or lymphadenopathy. His vaccination to date was complete as per the national immunization program. There was no similar family history.

**Figure 1:**
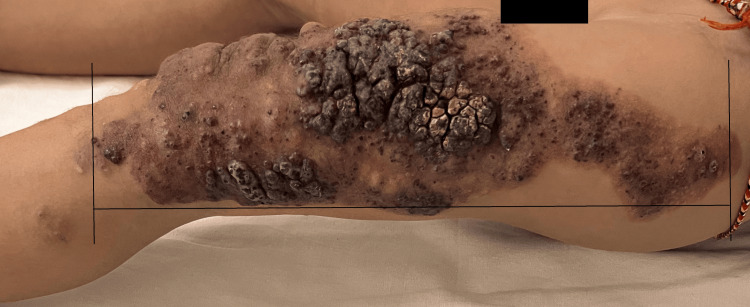
Multiple red to bluish-black papules and nodules that form a verrucous plaque over the port-wine stain

A detailed cutaneous examination revealed multiple red to bluish-black papules and nodules that formed a plaque with a verrucous surface on a large port wine stain of size 21 cm X 6 cm extending from the left mid-inguinal crease to the left knee (Figure 1).

**Figure 2:**
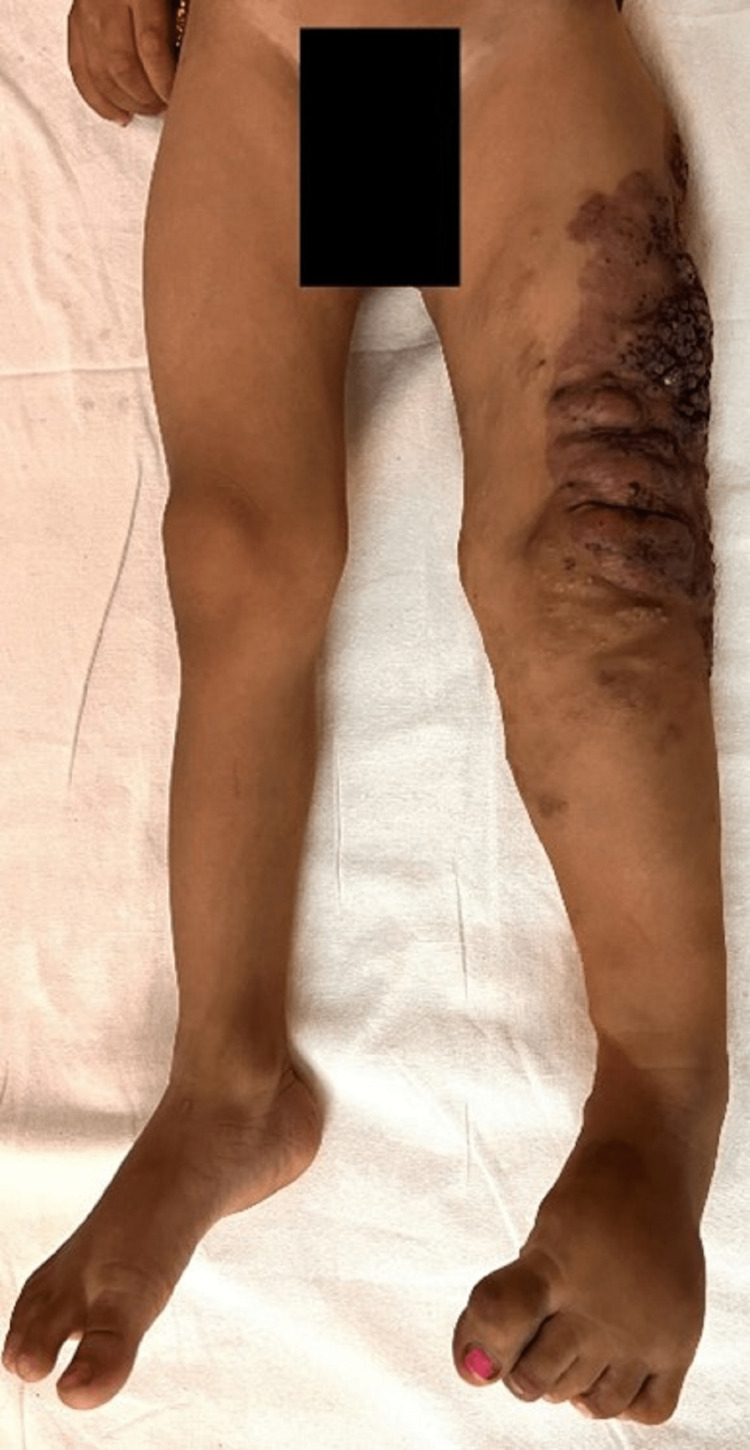
Gross hypertrophy of the left lower limb

**Figure 3:**
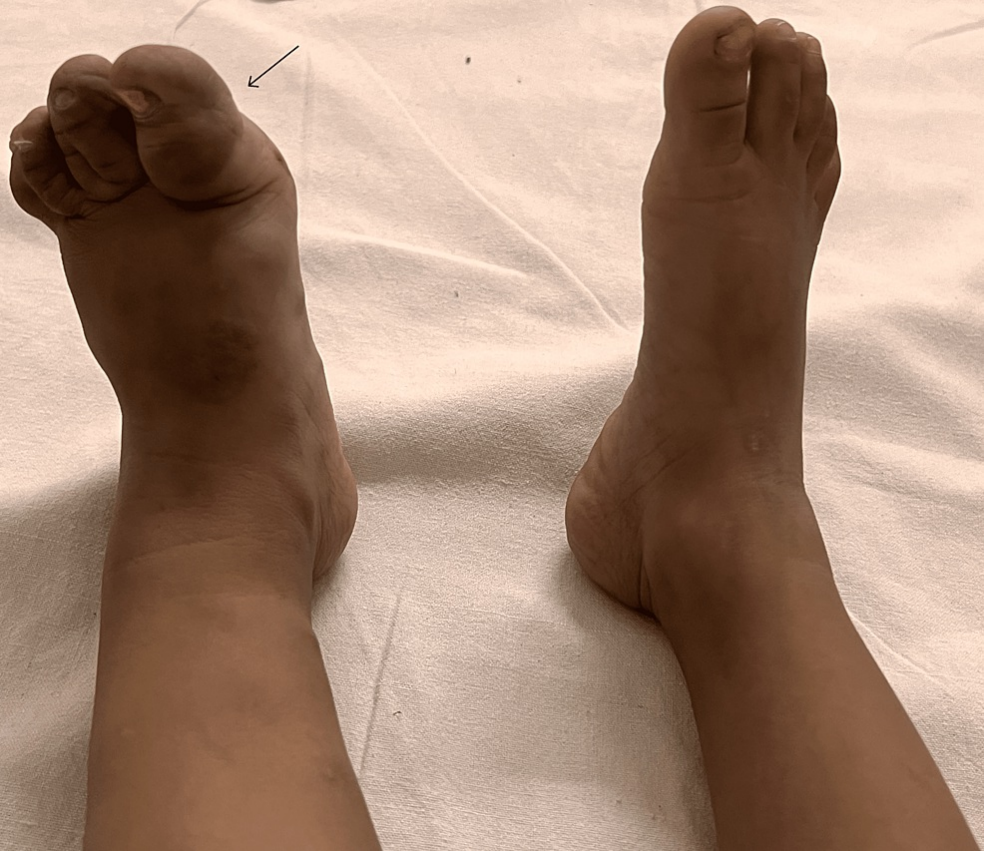
Macrodactyly of the left foot

He had gross hypertrophy of the left lower limb with measurable lengthening compared to the opposite limb. When measuring both the lower limb from the anterior superior iliac spine to the heel, the left lower limb was 2 cm longer and approximately 2.5 to 3.5 cm broader than the right lower limb throughout the length (Figure 2). The left foot had macrodactyly (Figure 3). His genitalia were normal.

Laboratory examinations revealed hemoglobin levels of 8.2 g/dL. Ultrasonography and color Doppler of the left limb revealed soft tissue hypertrophy with abnormal venous communication. The arterial tree appeared normal in caliber, with an echo-free lumen, and with a normal waveform without evidence of thrombosis. Timolol drops were prescribed as a topical application on the lesion for symptomatic relief and he was discharged after control of bleeding. Now, the patient is receiving routine follow-ups.

## Discussion

Hemangiectatic hypertrophy syndrome and angio-osteohypertrophy syndrome are other names for KTS. The first case was reported by Maurice Klippel and Paul Trénaunay in 1900 [7].

KTS has a sporadic incidence without any racial or sex predilection [8]. The incidence is approximately 1:100,000 births [1]. KTS is a disorder with unknown etiology; many hypotheses have been postulated including mesodermal abnormalities, somatic mosaicism, altered embryonic vasculogenesis, and paradominant inheritance, and some cases have been reported with different translocations and mutations, but the definitive association with the disease has not been proven. Translocation at (8;14) (q22.3;q13) [9], supernumerary ring chromosome 18 [10], terminal deletion 2q37.3 [11], and balanced translocation [12] are some examples. Currently, it is believed to be caused by somatic mosaicism in the phosphoinositide 3-kinase/PIK3CA signaling pathway [13]. KTS commonly presents as a classic clinical triad of limb hypertrophy (asymmetrical limb measurements with soft tissue overgrowth), varicosity, and vascular defects. The diagnosis of KTS is based on clinical examinations and imaging investigations [14]. Usually, findings are limited to one extremity, but can involve multiple extremities, and even the whole body can be involved, of which leg involvement is more commonly seen [15]. In approximately 20% of patients with KTS, involvement of the gastrointestinal tract is observed [16] and is associated with life-threatening symptom-free gastrointestinal bleeding [17].

The cutaneous capillary abnormality found in KTS is called a port wine stain which is non-blanchable, distributed dermatomal, and mostly unilateral. Cutaneous defects are commonly observed on the affected side of the limb [18]. Most of these vascular abnormalities are restricted to the skin, but can also affect the muscles and bones of the affected limb and can extend to affect organs such as the pleura, spleen, liver, bladder, and colon. This capillary malformation can show significant fading with age [15]. Most patients with KTS have hypertrophy of the bone and soft tissues that is clinically evident as increased length and girth of the affected limb. The increase in girth of the involved limb is primarily due to the overgrowth of soft tissue and may be contributed by muscular hypertrophy together with increased vascularity and skin thickness [18].

In general, the single lower limb is involved, but multiple limbs or partial hypertrophy of the single limb can be seen in KTS [19]. The varicosities seen in KTS are extensive and their development starts at a young age, as a child becomes ambulatory. The most prominent varicosity seen is a large incompetent vein on the lateral side of the affected leg. Approximately 68% to 80% of patients with KTS have a large lateral vein [18]. Skeletal malformations such as hip dislocation, syndactyly, and scoliosis can be seen in KTS, where hip dislocation is the most common, and it is believed to be secondary to limb length abnormalities [20]. Lymphatic anomalies associated with KTS such as lymphatic aplasia or hypoplasia can cause lymphedema [18]. Other proliferative lesions in children include 1) tumors of the surface epithelium (epidermal nevi), 2) melanocytic lesions, 3) tumors of the skin appendage/connective tissue (pilomatrixoma, dermatofibroma, etc.), 4) cutaneous cysts (epidermoid/trichilemmal cysts), 5) vascular lesions (lobular capillary, capillary, cavernous hemangioma, etc.), and 6) lymphoproliferative/related disorders [21]. 

The treatment of KTS is mainly symptomatic and should be approached conservatively if the patient has functional limbs, no edema, bleeding, ulceration, or pain. Duplex ultrasound has high sensitivity and specificity for vascular anatomy and function and is the best primary diagnostic test; many studies suggest MRI for diagnostic evaluation, as it provides better differentiation of the soft tissues [22]. Capillary malformations are treated only if they become symptomatic and can be treated with laser or sclerotherapy; some cases may require skin grafting. Lymphatic malformation can be treated as capillary malformation and rarely requires resection. The surgery aims to remove the varicose veins and insufficient perforators. Cavernous haemangiomas can lead to more or less serious bleeding, which requires surgical correction. Before any radiological or surgical intervention, heparin anticoagulation is often required, as there is the possibility of blood stasis in varicosities that can cause thrombosis. Sclerotherapy or endovascular lasers can be used to obliterate large venous channels. For vascular and lymphatic malformations, sirolimus is an emerging treatment option [23]. Complications such as deep vein thrombosis, hemorrhage, pulmonary embolism, gastrointestinal bleeding, stasis dermatitis, cellulitis, and limb hypertrophy can be seen in KTS [15].

## Conclusions

We present an interesting and rare lymphovascular congenital disorder, KTS, in a child from central India who presented papulonodular verrucous plaques on a large port wine stain and gross hypertrophy of the left lower limb. This case provides a review of the clinical presentation and etiopathogenesis of KTS. It usually has a slow and benign clinical course; multidisciplinary management with adequate conservative and surgical intervention provides a normal routine life to affected patients, and regular follow-ups help prevent the development of complications, which can alter the prognosis of the disease.
